# Fluorometric Detection of Low-Abundance EGFR Exon 19 Deletion Mutation Using Tandem Gene Amplification

**DOI:** 10.4014/jmb.2004.04010

**Published:** 2020-04-24

**Authors:** Dong-Min Kim, Shichen Zhang, Minhee Kim, Dong-Eun Kim

**Affiliations:** Department of Bioscience and Biotechnology, Konkuk University, Seoul 05029, Republic of Korea

**Keywords:** epidermal growth factor receptor, G-quadruplex, In-frame deletion mutation, rolling circle amplification, thioflavin T

## Abstract

Epidermal growth factor receptor (EGFR) mutations are not only genetic markers for diagnosis but also biomarkers of clinical-response against tyrosine kinase inhibitors (TKIs) in non-small cell lung cancer (NSCLC). Among the EGFR mutations, the in-frame deletion mutation in EGFR exon 19 kinase domain (EGFR exon 19-del) is the most frequent mutation, accounting for about 45% of EGFR mutations in NSCLCs. Development of sensitive method for detecting the EGFR mutation is highly required to make a better screening for drug-response in the treatment of NSCLC patients. Here, we developed a fluorometric tandem gene amplification assay for sensitive detection of lowabundance EGFR exon 19-del mutant genomic DNA. The method consists of pre-amplification with PCR, thermal cycling of ligation by *Taq* ligase, and subsequent rolling circle amplification (RCA). PCRamplified DNA from genomic DNA samples was used as splint DNA to conjugate both ends of linear padlock DNA, generating circular padlock DNA template for RCA. Long stretches of ssDNA harboring multiple copies of G-quadruplex structure was generated in RCA and detected by thioflavin T (ThT) fluorescence, which is specifically intercalated into the G-quadruplex, emitting strong fluorescence. Sensitivity of tandem gene amplification assay for detection of the EGFR exon 19-del from gDNA was as low as 3.6 pg, and mutant gDNA present in the pooled normal plasma was readily detected as low as 1% fraction. Hence, fluorometric detection of low-abundance EGFR exon 19 deletion mutation using tandem gene amplification may be applicable to clinical diagnosis of NSCLC patients with appropriate TKI treatment.

## Introduction

Lung cancer is one of the high mortality cancers, accounting for about 18.4% of the total cancer deaths, with around 18% of five-year survival rate [1]. Among various types of lung cancer, non-small cell lung cancer (NSCLC) patients comprise over 80% of all lung cancer cases [2]. Epidermal growth factor receptor (EGFR) mutations that are generally observed in the NSCLCs are thus genetic markers for diagnosis of NSCLC. Mutations in EGFR genes are also associated with response of tyrosine kinase inhibitors (TKIs), such as gefitinib, erlotinib, and afatinib, which have been standard provision for NSCLC patients [3]. Various EGFR mutations can alter the clinical response of EGFR-TKIs by changing the drug binding affinity to target region in EGFR [4, 5]. One of the most frequent EGFR kinase domain mutations is the in-frame deletion mutation in exon 19 (*i.e.*, EGFR exon 19-del), accounting about 45% of EGFR mutations in NSCLCs [6]. Thus, a sensitive and accurate method for detecting the in-frame deletion EGFR mutation is highly required to make a better screening for drug-response in the treatment of NSCLC patients.

Although tissue samples have been widely used to identify EGFR mutations, small proportion of mutant cancer cells is present at a low-abundance in clinically available tissue samples. Recently, instead of tissue biopsy sampling, circulating tumor DNA (ctDNA) in plasma has emerged as a reliable biomarker for detecting somatic mutations due to the advantages such as less-invasiveness and reflection of whole information of heterogenous tumor [7]. To date, several methods including Sanger DNA sequencing, next generation sequencing (NGS), and real-time PCR with TaqMan probe have been considered as a standard method for monitoring EGFR mutations [8, 9]. Alternatively, there are various PCR-based modifications using DNA-LNA blocker [10], universal oligo-quencher [11], allele-specific probe [12], and graphene oxide [13]. Despite several PCR-based modifications have been developed in an attempt to detect ctDNA, they still have limitations such as low sensitivity for detecting low frequency of mutant ctDNA in the plasma.

One of potential approaches to overcome shortcomings in PCR-based gen amplification methods is to improve sensitivity by combining two consecutive gene amplification methods such as PCR and isothermal gene amplification. Rolling circle amplification (RCA) is an effective isothermal amplification method that can be coupled with the other amplification methods [14-16]. The RCA reaction product, which is long stretch of single-stranded DNA (ssDNA) harboring tandem sequences generated by high fidelity of phi29 polymerase, can be simply detected by G-quadruplex (GQ) and thioflavin T (ThT)-based fluorometric system [17]. In this system, ThT can selectively intercalate into the four-stranded DNA structure of G-quadruplex and emit strong fluorescence with high signal to background (S/B) ratio [18]. Although there have been several attempts to combine RCA with PCR-amplified amplicon to improve sensitivity, their application is still limited due to the fact that single-stranded oligonucleotide is usually required for ligation of padlock DNA template for RCA initiation.

In this study, to overcome the uneasy preparation of ssDNA to initiate subsequent RCA, we developed a highly sensitive and selective detection method for low-abundance EGFR exon 19 deletion mutation (exon 19-del) in plasma using tandem gene amplification methods. The method consists of pre-amplification with PCR, thermal cycling of ligation by *Taq* ligase, and RCA by phi29 DNA polymerase. Taking advantage of high selectivity of *Taq* ligase at high temperature and fidelity of phi29 DNA polymerase, circularization of the padlock DNA and subsequent ThT fluorescence from tandem GQ-containing RCA product were only obtained in the presence of mutant genomic DNA (gDNA) harboring the EGFR exon 19-del. In addition, our fluorometric assay was applied to detect mutant gDNA in plasma, which provided detection limit of 1% in the mixture with wild-type gDNA. This accurate, sensitive, and UV-visualizable fluorometric assay can be applicable for clinical diagnosis of the EGFR exon 19-del to provide proper information for a better TKI prescription.

## Materials and Methods

### Reagents, Oligonucleotides, and Lung Cancer Cells

DNA oligonucleotides including PCR primers and padlock probe DNA were chemically synthesized and purified by high performance liquid chromatography (Bionics, Korea). The padlock probe DNA that can hybridize to the sequence of EGFR exon 19-del site was modified with phosphate at the 5’-end to be ligated into a circular template for subsequent RCA. The sequences of the oligonucleotides are listed in Table S1. Ex *Taq* polymerase and dNTPs mixture (dATP, dGTP, dCTP, and dTTP) was purchased from Takara Korea Biomedical Inc. (Korea). Thioflavin T (ThT) was purchased from Sigma-Aldrich Korea (Korea). Phi29 DNA polymerase and *Taq* DNA ligase were purchased from New England Bio-labs (USA). Pooled normal human plasma was purchased from Innovative Research (Novi, MI, USA).

The A549 cell line was from the American Type Culture Collection (ATCC, CCL-185, USA) and PC9 cell line was from the European Collection of Authenticated Cell Cultures (ECACC, 90071810, UK). A549 cells possess the wild-type EGFR exon 19 gene, whereas PC9 cells contain EGFR exon 19 deletion mutation (E746-A750). All cells were cultured in DMEM high glucose (Hyclone, USA) supplemented with 10% fetal bocine serum (Hyclone) and 100 U/ml penicillin (WelGene, Korea) in a humidified incubator under 5% CO_2_ at 37°C.

### Genomic DNA Extraction and PCR

The genomic DNA (gDNA) from A549 and PC9 was extracted with QIAamp DNA Mini Kit (Qiagen, Germany) following the manufacturer’s instructions and 50 ng of extracted gDNAs was used for PCR. The PCR mixture (20 μl) was composed of 0.5 μl of Ex *Taq* polymerase (2.5 U), 1× Ex *Taq* buffer, 0.2 mM dNTP, and 300 nM of PCR primers. PCR was carried out by following conditions: heat denaturation at 95°C 3 min, subsequent 35 cycles (95°C for 30 sec, 61°C for 30 sec, 68°C for 30 sec), and 68°C for 3 min. The PCR products were analyzed and identified by 2% agarose gel electrophoresis under UV illumination after ethidium bromide staining.

### Circularization of Padlock DNA

Formation of closed circular padlock probe was accomplished by *Taq* DNA ligase using a 2 μl aliquot of the PCR product or 1 μM of synthesized DNA harboring the wild-/mutant type sequence of EGFR in a reaction mixture (10 μl) containing 40 U of *Taq* ligase, 1× *Taq* ligase buffer, and 1 μM of padlock probe DNA. The ligation was conducted under following conditions: pre-denaturation of PCR product at 95°C for 5 min, 15 cycles of thermal ligation reaction (denaturation at 95°C for 30 sec and ligation at 60°C for 5 min), and heat inactivation at 95°C for 15 min. To examine whether the closed circular padlock probe was specifically generated through ligation, 10 μl of the ligation product was digested in the mixture (15 μl) containing 1× exonuclease I reaction buffer, 20 U exonuclease I (New England Biolabs), and 100 U exonuclease III (New England Biolabs). After incubation at 37°C for 2 h and subsequent heat inactivation at 95°C for 10 min, the digested products were analyzed by 12% Urea PAGE gel electrophoresis with SYBR gold (Life Technologies, USA).

### Fluorogenic Rolling Circle Amplification

The fluorogenic RCA was performed in 25 μl of RCA mixtures containing 100 nM circular padlock probe, 100 nM RCA primer, 1× phi29 polymerase buffer, 15 μM ThT, 0.5 mM dNTPs, 50 mM KCl, and 173U phi29 polymerase. The mixture was incubated at 32°C for 60 min and heat inactivated at 65°C for 15 min. After adding 25 μl of DW to RCA product, the ThT fluorescence was scanned from 450 nm to 650 nm using a Cary Eclipse Fluorescence Spectrophotometer (Agilent Technologies, USA) with excitation wavelength of 430 nm. To evaluate the sensitivity of PCR coupled ligation-mediated RCA assay for detecting EGFR exon 19 deletion mutation, the assay was performed with decreasing amounts of PC9 gDNA from 25 ng to 6 pg. The limit of detection (LOD) was determined by the following equation: LOD = 3.3 × standard deviation of the fluorescence intensity, SD/slope of the standard curve, S) based on the average signal of blank experiments and standard deviation obtained from triplicate measurements. To mimic the clinical sample, a fixed amount of gDNA mixture (2,000 ng) containing different mixing ratios (0-100% in mutant type gDNA) of A549 gDNA (wild-type) and PC9 gDNA (mutant type) was spiked into 100 μl of pooled normal human plasma. After gDNA was extracted using the QIAamp DNA mini kit and resolved in 100 μl, the 10 μl aliquot was used for the PCR coupled ligation-mediated RCA assay.

### Quantitative Real-Time PCR

To compare the sensitivity for detection of EGFR exon 19 deletion mutation, conventional quantitative real-time PCR (qRT-PCR) was performed with the same PCR primers used for the PCR coupled ligation-mediated RCA assay. Decreasing amounts of PC9 gDNA from 25 ng to 6 pg were subjected to PCR in the 20 μl mixture containing 0.5 μl of Ex *Taq* polymerase (2.5 U), 1× Ex *Taq* buffer, 0.2 mM dNTP, 300 nM of PCR primers, and 1× SYBR green. The qRT-PCR was carried out under same conditions as described above (Genomic DNA extraction and PCR section)

## Results and Discussion

### Tandem Gene Amplification Strategy

**Fig. 1:**
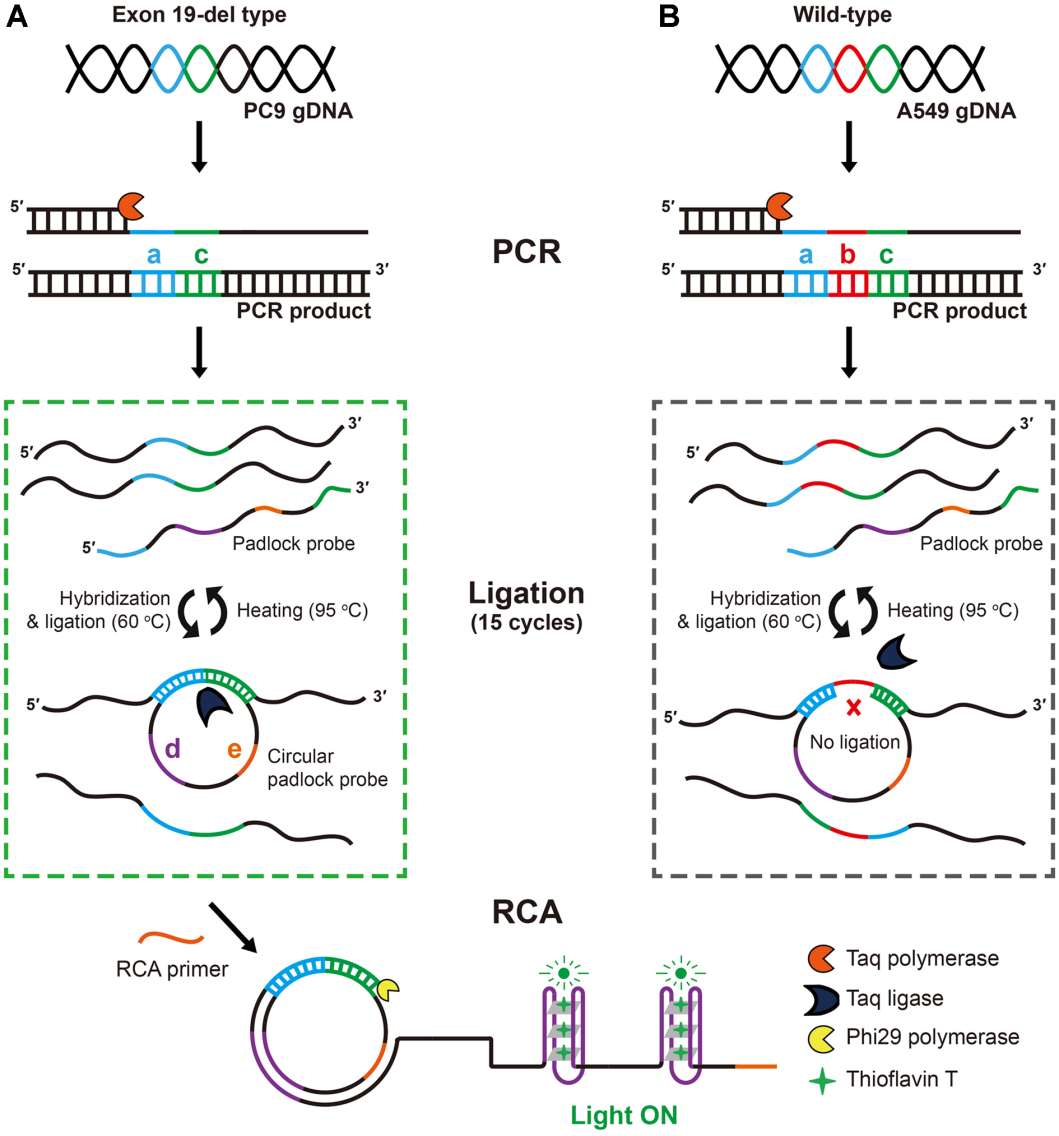
Schematic illustration of tandem gene amplification for detection of the EGFR exon 19 deletion mutation. Segment a and c: adjacent sequences of the deletion site; segment b: deletion mutation site; segment d: Gquadruplex sequence; segment e: RCA primer.

To achieve highly sensitive and accurate detection of the EGFR exon 19-del mutant conferring different clinical response against EGFR-TKIs in NSCLC patient, we designed a fluorometric method consisting of three steps: i) PCR of gDNA as a pre-amplification, ii) circularization of padlock DNA by *Taq* ligase cycling reaction, and iii) subsequent GQ-RCA (Fig. 1). In the pre-amplification of PCR, both EGFR genes harboring wild-type sequence with 15-nucleotides (nts) of deletion site (segment b) or mutant type sequence without the 15-nts segment b are amplified, resulting in accumulation of 109 bp or 94 bp of duplex DNA amplicons, respectively. During the 15 thermal cycles of *Taq* ligase reaction, both ends of padlock DNA are hybridized to the adjacent sequences (segment a and c) of the deletion site in both types of amplicon. Importantly, both ends of the padlock DNA are juxtaposed close enough to be ligated by *Taq* DNA ligase only when hybridized with a mutant type sequence. After the 15 cycles of ligation, RCA that is triggered by adding a RCA primer (segment e) and phi29 polymerase generates long stretch of ssDNA products containing multiple copies of G-quadruplex (segment d). Upon the ThT intercalating into GQ structure, ThT fluorescence is generated and quantified for presence of mutant DNA. In the absence of mutant gDNA (*i.e.*, wild-type gDNA), both ends of padlock DNA are not ligated because they are positioned far apart due to the intervening 15-nts of deletion site sequence.

**Fig. 2:**
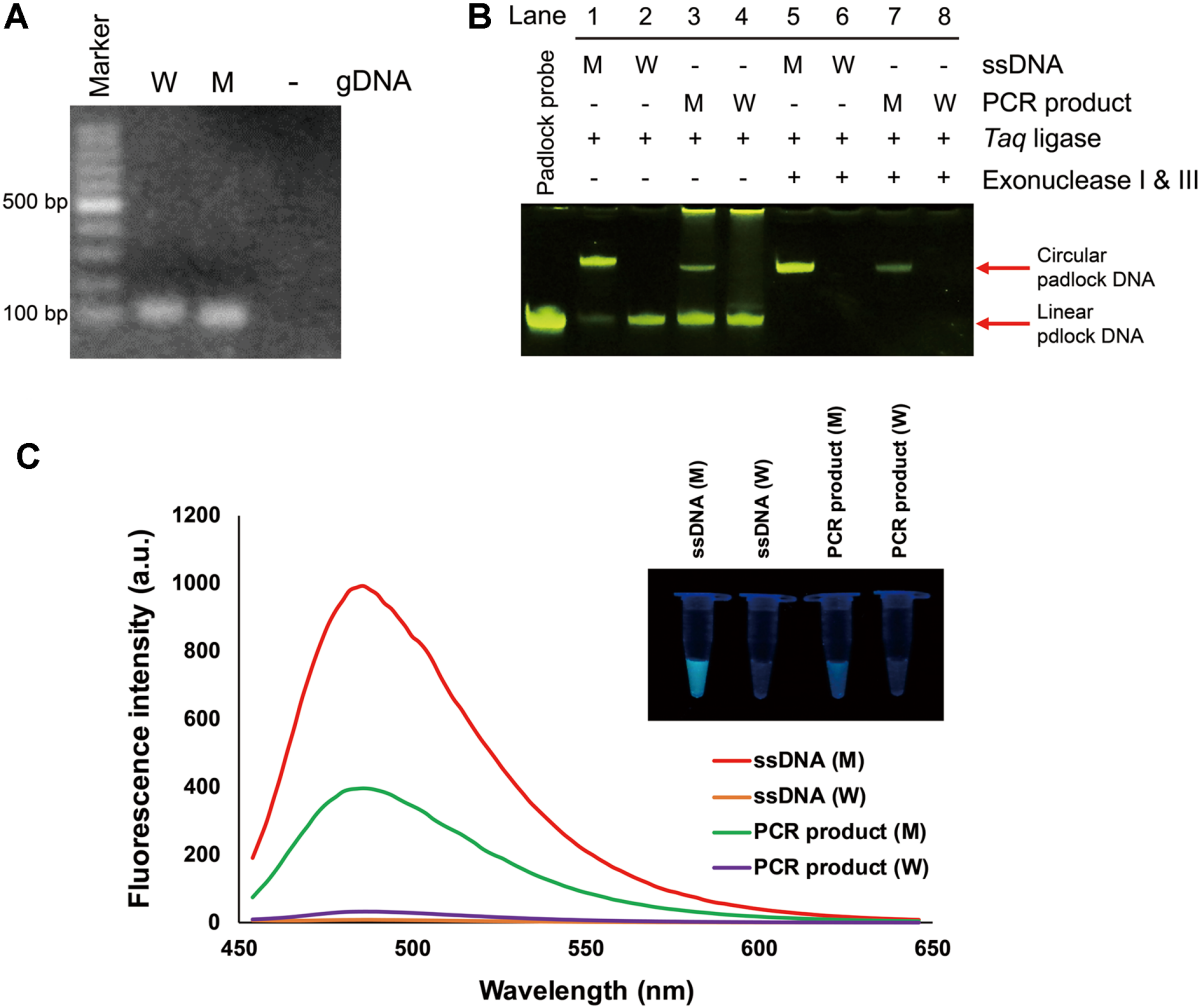
Evaluation of tandem gene amplification with ThT fluorescence enhancement. (**A**) Agarose gel (2%) electrophoresis showing the PCR products amplified with A549 gDNA (wild-type, W) or PC9 gDNA (mutant type, M). Marker lane is 100 bp DNA ladder. (**B**) Target mutant gene-specific circularization of padlock DNA. Linear padlock DNA was hybridized and ligated by *Taq* ligase with synthesized ssDNA or PCR-amplified dsDNA amplicon containing wild-type or mutant type. After degradation by exonuclease I & III, digested products were resolved by denaturing 12% urea-PAGE and stained with SYBR Gold. (C) Fluorescence emission spectra of ThT (λ_ex_ = 430 nm) obtained with synthesized ssDNA or PCRamplified dsDNA amplicon containing wild-type or mutant type. Inset image showed the RCA product visualized under UV light.

To validate the eligibility of the tandem gene amplification assay, we first analyzed the PCR products amplified with A549 and PC9 gDNA containing wild-type and exon 19-del mutation (Fig. 2A). The amplicon with A549 gDNA was observed at a slightly higher position than amplicon with PC9 gDNA, which is consistent with the expected sizes of 109 bp for wild-type and 94 bp for mutant type. Next, we examined the circularization of padlock DNA complementary to sequence of the deletion site (Fig. 2B). Synthesized ssDNA and PCR-amplified dsDNA containing wild-type or mutant type sequence of EGFR were used as splint DNAs for cycling ligation with the padlock DNA. The circular padlock DNAs were only observed in the presence of mutant type DNAs (lane 1 and 3). Furthermore, the closed circular padlock DNA remained intact after treatment of exonuclease I & III, which can digest ssDNA and dsDNA with exposed ends, respectively (lane 5 and 7). In contrast, the padlock DNAs ligated with wild-type DNAs were fully degraded by exonuclease I & III (lane 6 and 8). This result demonstrates that the target mutant DNA sequence can be used as a proper splint DNA to conjugate both ends of padlock DNA. Next, we evaluated the G-quadruplex generating RCA and ThT fluorescence enhancement, in which the RCA product of ssDNA harboring multiple copies of GQ structure was monitored by ThT fluorescence. Increase in the ThT fluorescence of RCA product was obtained from both mutant-type sequence of ssDNA and dsDNA amplicon (Fig. 2C). The fold-increase in fluorescence intensity at 488 nm between RCA product with mutant and wild-type were calculated to be 92.8 (ssDNA) and 11.4 (dsDNA amplicon). The bright blue fluorescence with mutant type gene was visualized under UV light, consistent with fluorescence spectra. These results clearly show that circularization of padlock DNA and subsequent GQ-RCA with ThT occurs only in the presence of target mutant gDNA. To explore the optimal number of cycles and temperature for *Taq* ligase reaction, the linear padlock DNA was subjected to ligation with PCR product containing wild-type (A549) or mutant type (PC9) sequence under different cycles (15, 25, and 35 cycles) (Fig. S1) or different temperature (55, 60, 65, and 70°C) (Fig. S2). Optimal number of cycles and temperature were found to be 15 cycles and 60 °C, respectively, with a maximal S/B ratio between mutant and wild-type.

### Sensitivity and Selectivity of Tandem Gene Amplification Assay

To examine the sensitivity of tandem gene amplification assay, decreasing amounts of PC9 gDNA (50 ng–6 pg) were used for the tandem gene amplification and scanned for fluorescence emission spectra of product. The ThT fluorescence intensity gradually decreased as the amount of PC9 gDNA decreased from 50 ng to 6 pg. The titration curve of fluorescence intensity at 488 nm obtained from the spectra showed a linear relationship between fluorescence intensity and the amount of PC9 gDNA with a calculated LOD value of 3.6 pg. Under UV light, the bright blue ThT fluorescence gradually increased as the amount of mutant gDNA increased, which is consistent with the titration curve.

**Fig. 3:**
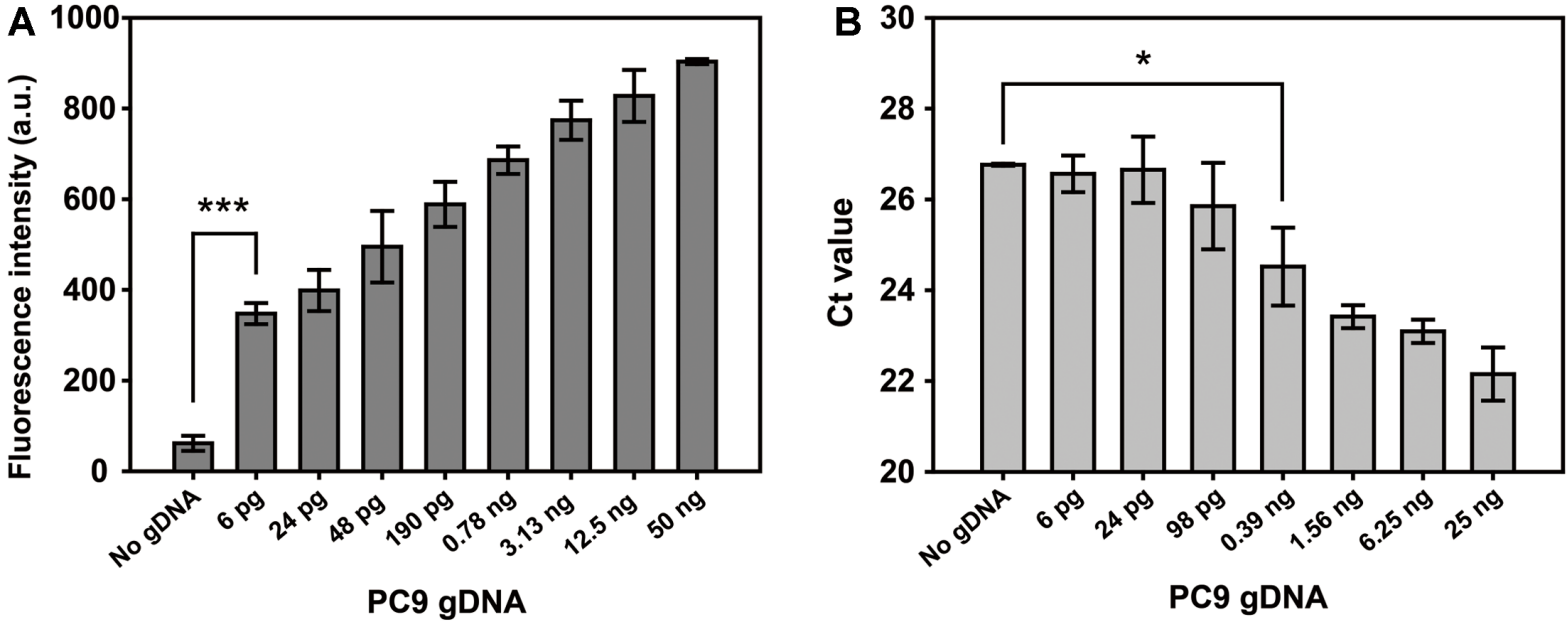
Sensitivity of tandem gene amplification assay. (**A**) Bar graphs represent fluorescence intensities obtained by tandem gene amplification for EGFR exon 19-del detection. The assay indicates a statistically significant positive detection at amount of mutant PC9 gDNA as low as 6 pg (***, *p* < 0.005 vs. No gDNA). (**B**) Quantitative real-time PCR for EGFR exon 19-del detection. Bar graphs represent the threshold cycle (Ct) values obtained during real-time PCR with decreasing amount of PC9 gDNA. The real-time PCR shows statistically significant positive detection at an amount of mutant PC9 gDNA as low as 0.39 ng (*, *p* < 0.05 vs No gDNA). The data are presented as the mean ± standard deviation of three experiments.

Next, we compared sensitivity of our assay with conventional real-time PCR based on SYBR green intercalating dye method. As shown in Fig. 3A, the fluorescence intensity from tandem gene amplification assay was statistically significant at amount of PC9 gDNA as low as 6 pg. The samples with decreasing amount of PC9 gDNA were analyzed using quantitative real-time PCR with SYBR green (Fig. 3B). The cycle threshold (Ct) values that were gradually increased as the amount of PC9 gDNA decreases showed an LOD as low as 0.39 ng. These data indicate that our system had a 65-fold higher sensitivity than SYBR green-based quantitative real-time PCR for detection of the EGFR in-frame deletion mutation. In addition, the selectivity of tandem gene amplification assay was evaluated with gDNA mixture containing different fractions of PC9 mutant gDNA (0 to 100%) in a fixed amount of total gDNA (50 ng). The ThT fluorescence intensity at 488 nm provided a statistically significant LOD as low as 0.1%, and the blue colored ThT fluorescence gradually increased as the fractions of mutant gDNA increased under UV light (Fig. S3). Thus, tandem gene amplification followed by ThT fluorescence enhancement provides a superior sensitivity to conventional real-time PCR for detection of the EGFR in-frame deletion mutant present in genomic DNA sample.

### Detection of the EGFR In-Frame Deletion Mutant gDNA in Blood Plasma

**Fig. 4:**
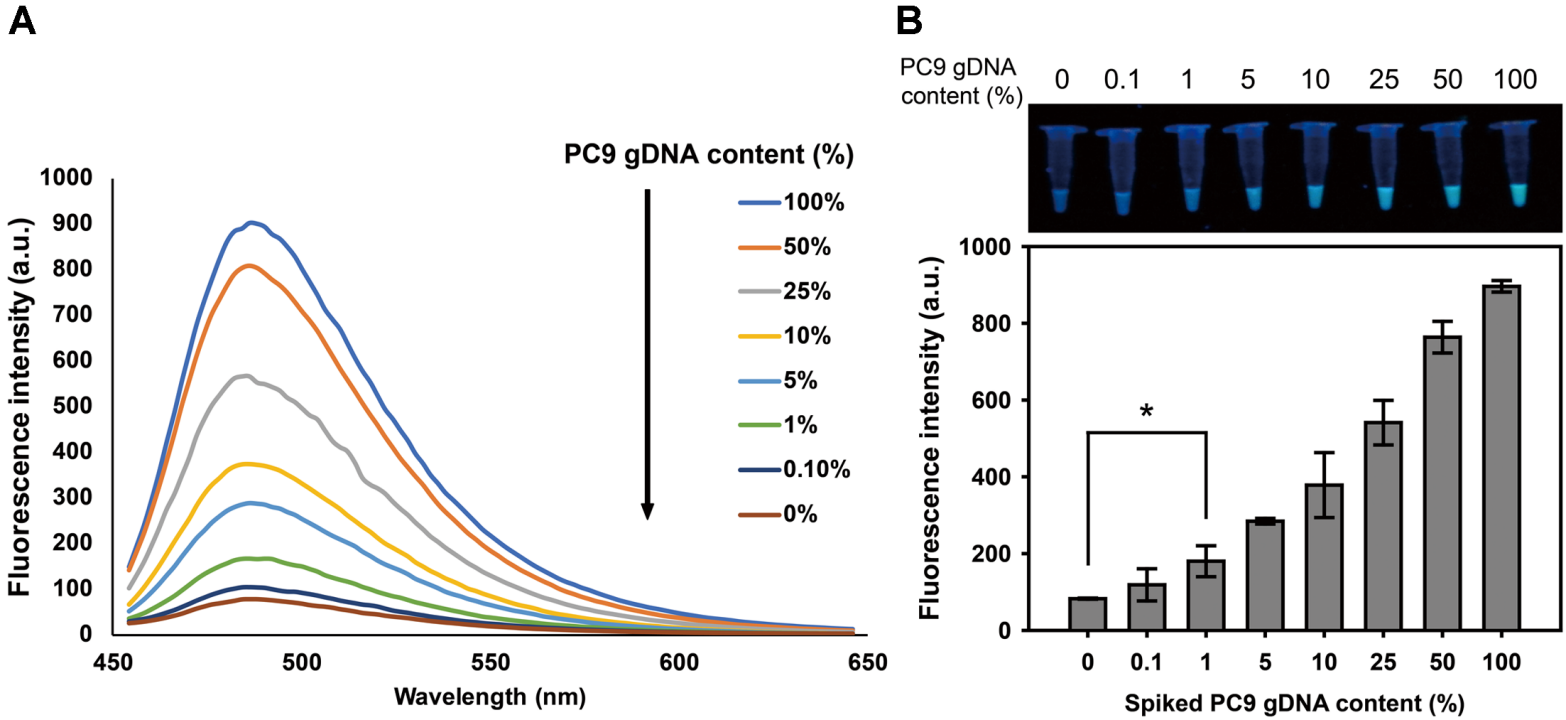
Quantitative detection of EGFR 19-del mutant genes spiked in the pooled human plasma. (**A**) Fluorescence emission spectra of ThT (λ_ex_ = 430 nm) obtained at different fractions of mutant gDNA in a fixed amount of gDNA mixture (2.0 μg), which were spiked in plasma (100 μl). (**B**) Bar graphs represent fluorescence intensities obtained at different fractions of mutant gDNA. The assay indicates a statistically significant positive detection at amount of mutant PC9 gDNA as low as 1% fraction (*, *p* < 0.05 vs. 0%). The data are presented as the mean ± standard deviation of three experiments. Inset image showed the RCA product visualized under UV light.

We further tested the detection capacity of our assay by using pooled normal human blood plasma samples (100 μl) spiked with gDNA mixture containing different proportions of mutant PC9 gDNA (0 to 100% in PC9 content) in a fixed amount of total gDNA (2.0 mg). After extraction of gDNA from blood plasma, 10 μl of gDNA was analyzed by the tandem gene amplification and the emission spectra of ThT fluorescence was scanned (Fig. 4A). The ThT fluorescence signal at 488 nm showed a linear correlation with the amount of PC9 gDNA and the LOD was found to be 1% (Fig. 4B). This result suggests that small numbers of mutant gDNA harboring the EGFR exon 19-del with abundant background of wild-type gDNA in plasma was able to be readily detected with our tandem gene amplification method.

In conclusion, we have developed a sensitive fluorometric assay for detection of EGFR exon 19-del mutant present in genomic DNA samples using the tandem gene amplification method. Stringency of *Taq* DNA ligase reaction resulted in high selectivity for distinguishing the target mutant gDNA from wild-type gDNA. Sensitivity of our tandem gene amplification assay for detection of the EGFR exon 19-del from gDNA was as low as 3.6 pg and mutant gDNA mixed with abundant wild-type gDNA was as low as 0.1%. Furthermore, mutant gDNA present in the pooled normal plasma as low as 1% fraction was readily detected with the method. Thus, fluorometric detection of low-abundance EGFR exon 19 deletion mutation using tandem gene amplification may be applicable to clinical diagnosis with a hope for appropriate prescription for NSCLC patient.

## Supplemental Materials



Supplementary data for this paper are available on-line only at http://jmb.or.kr.

## References

[1] Siegel RL, Miller KD, Jemal A (2017). Cancer Statistics, 2017. CA Cancer J. Clin..

[2] Sharma SV, Bell DW, Settleman J, Haber DA (2007). Epidermal growth factor receptor mutations in lung cancer. Nat. Rev. Cancer.

[3] Herbst RS, Morgensztern D, Boshoff C (2018). The biology and management of non-small cell lung cancer. Nature.

[4] Lynch TJ, Bell DW, Sordella R, Gurubhagavatula S, Okimoto RA, Brannigan BW (2004). Activating mutations in the epidermal growth factor receptor underlying responsiveness of non-small-cell lung cancer to gefitinib. New Engl. J. Med..

[5] Tokudome N, Koh Y, Akamatsu H, Fujimoto D, Okamoto I, Nakagawa K (2020). Differential significance of molecular subtypes which were classified into EGFR exon 19 deletion on the first line afatinib monotherapy. BMC Cancer.

[6] Mitsudomi T, Yatabe Y (2010). Epidermal growth factor receptor in relation to tumor development: EGFR gene and cancer. FEBS J..

[7] Schwarzenbach H, Hoon DSB, Pantel K (2011). Cell-free nucleic acids as biomarkers in cancer patients. Nat. Rev. Cancer.

[8] Zhang Y, Shen WX, Zhou LN, Tang M, Tan Y, Feng CX (2020). The value of next-generation sequencing for treatment in nonsmall cell lung cancer patients: The observational, real-world evidence in China. Biomed. Res. Int..

[9] Yatabe Y, Hida T, Horio Y, Kosaka T, Takahashi T, Mitsudomi T (2006). A rapid, sensitive assay to detect EGFR mutation in small biopsy specimens from lung cancer. J. Mol. Diagn..

[10] Ren XD, Liu DY, Guo HQ, Wang L, Zhao N, Su N (2019). Sensitive detection of low-abundance in-frame deletions in EGFR exon 19 using novel wild-type blockers in real-time PCR. Sci. Rep..

[11] Bae JH, Jo SM, Kim HS (2015). Comprehensive detection of diverse exon 19 deletion mutations of EGFR in lung cancer by a single probe set. Biosens. Bioelectron..

[12] Weber B, Meldgaard P, Hager H, Wu L, Wei W, Tsai J (2014). Detection of EGFR mutations in plasma and biopsies from nonsmall cell lung cancer patients by allele-specific PCR assays. BMC Cancer.

[13] Kim DM, Kim DH, Jung W, Lee KY, Kim DE (2018). Fluorometric detection of EGFR exon 19 deletion mutation in lung cancer cells using graphene oxide. Analyst.

[14] Blanco L, Bernad A, Lazaro JM, Martin G, Garmendia C, Salas M (1989). Highly efficient DNA synthesis by the phage phi 29 DNA polymerase. Symmetrical mode of DNA replication. J. Biol. Chem..

[15] Goo NI, Kim DE (2016). Rolling circle amplification as isothermal gene amplification in molecular diagnostics. Biochip J..

[16] Kim DM, Seo J, Jun BH, Kim DH, Jeong W, Hwang SH (2017). Fluorometric detection of influenza virus RNA by PCR-coupled rolling circle amplification generating G-quadruplex. Sensor. Actuat. B-Chem..

[17] Fujita H, Kataoka Y, Tobita S, Kuwahara M, Sugimoto N (2016). Novel One-Tube-One-Step real-time methodology for rapid transcriptomic biomarker detection: signal amplification by ternary initiation complexes. Anal. Chem..

[18] Mohanty J, Barooah N, Dhamodharan V, Harikrishna S, Pradeepkumar PI, Bhasikuttan AC (2013). Thioflavin T as an efficient inducer and selective fluorescent sensor for the human telomeric g-quadruplex DNA. J. Am. Chem. Soc..

