# Supplementary material for: Fluorometric Detection of Low-Abundance EGFR Exon 19 Deletion Mutation Using Tandem Gene Amplification
Source: J Microbiol Biotechnol. 2020 Apr 24;30(5):662–7. doi: 10.4014/jmb.2004.04010 (PMC9728277; doi:10.4014/jmb.2004.04010)
Supplement: Supplementary file 1 [file JMB-30-5-662-supple.pdf]

J. Microbiol. Biotechnol.
https://doi.org/10.4014/jmb.2004.04010

Supplementary data

Table S1. Oligonucleotide sequences (5' to 3') used in the assay.

Name	Sequence* (5′ → 3′)	Size* (nt)	PCR amplicon size (bp)
EGFR forward primer	GACTCTGGATCCCAGAAGGTGA	22	109 for wild-type<br>94 for mutant type
EGFR reverse primer	CAGAAACTCACATCGAGGATTTCC	24	
Padlock probe DNA	p-TTGATAGCGACGGGATATTTTGCACTTAACTATACTAATCCAA<br>CCCTAACCCTAACCCTAACCCTATTTGCTTTCGGAGATGTT	84	
RCA primer	GGATTAGTATAGTTAAGTGC	20	

* Target recognition site near EGFR exon 19-del site, cyan- and green-colored segment; RCA primer binding site, orange-colored segment; G-quadraplex generating sequence, purple-colored segment; p, phosphorylation; nt, nucleotides.

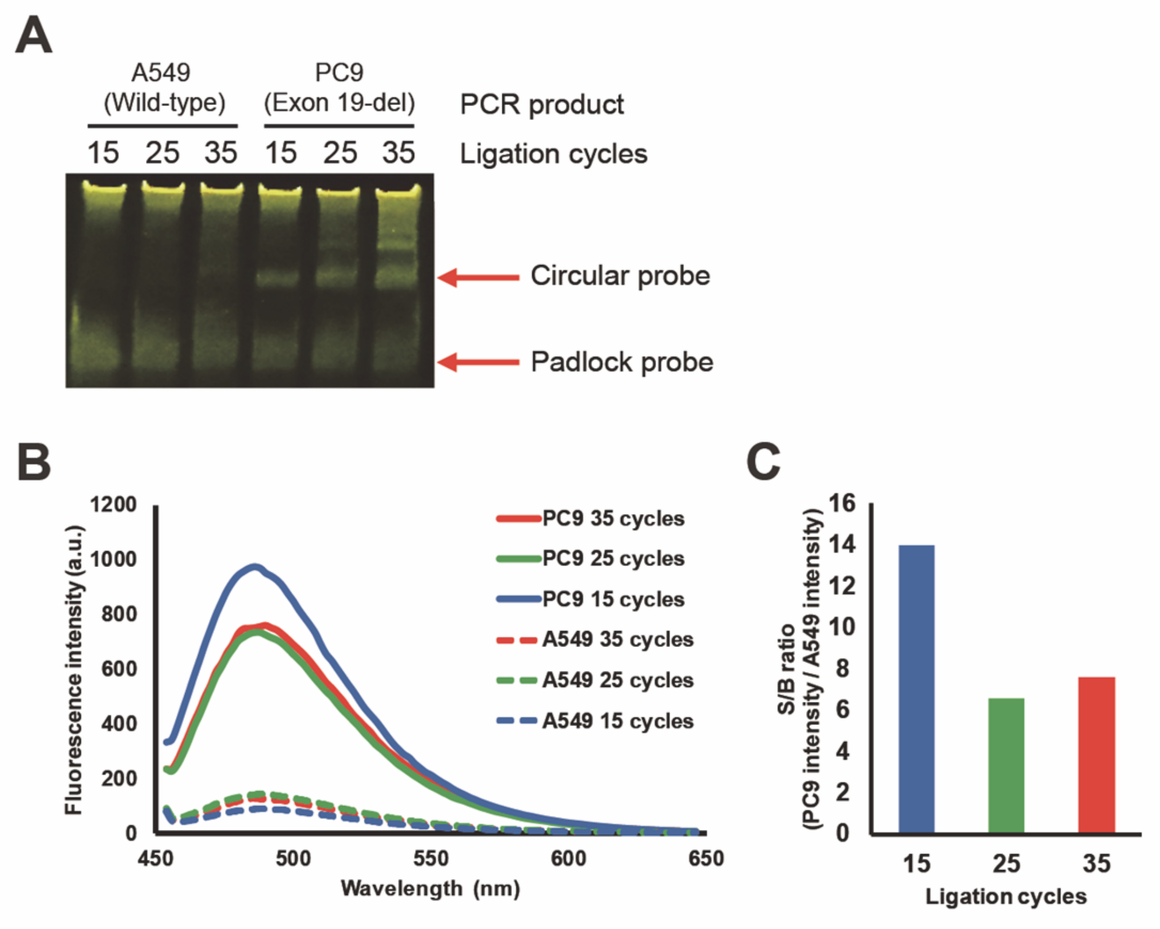

Fig S1. Optimization of number of cycles for thermal ligation reaction. (A) Circularization of padlock DNA with different number of cycles was analyzed by denaturing 12% urea-PAGE. After staining by SYBR Gold, the circular probe DNA bands were observed only in the presence of PCR product harboring mutant sequence (EGFR exon 19-del). (B) Fluorescence emission spectra of ThT (λ_{ex} = 430 nm) obtained at different cycles of ligation. (C) Bar graphs represent signal to background ratio (S/B ratio) between fluorescence intensity of PC9 and that of A549 at 488 nm. The maximal S/B ratio was observed with 15 cycles of thermal ligation.

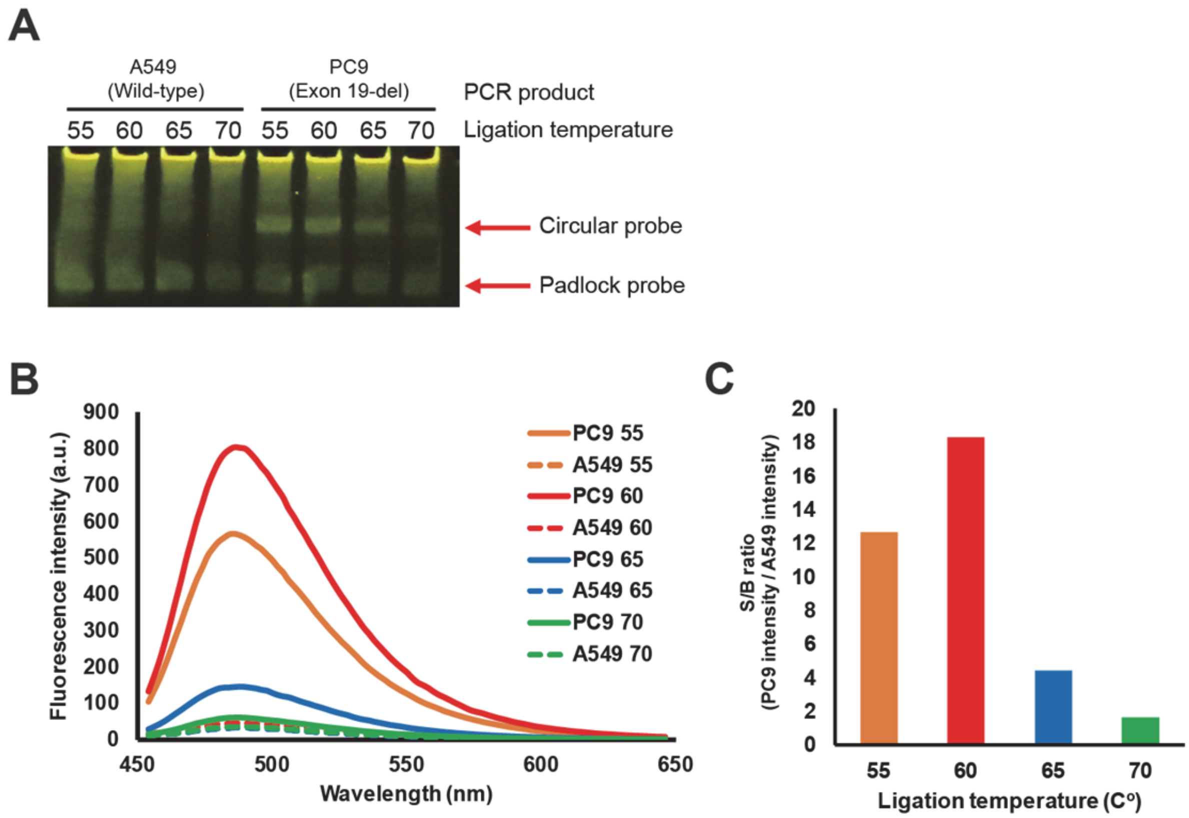

Fig S2. Optimization of the reaction temperature for ligation reaction. (A) Circularization of padlock DNA with different temperature was analyzed by denaturing 12% urea-PAGE. After staining by SYBR Gold, the circular probe DNA bands were observed only in the presence of PCR product harboring the mutant sequence (EGFR exon 19-del). (B) Fluorescence emission spectra of ThT (λ_{ex} = 430 nm) obtained at different temperature. (C) Bar graphs represent S/B ratio between fluorescence intensity of PC9 and that of A549 at 488 nm. The reaction temperature was optimized to maximize the S/B ratio and it was found to be 60 ^{o}C.

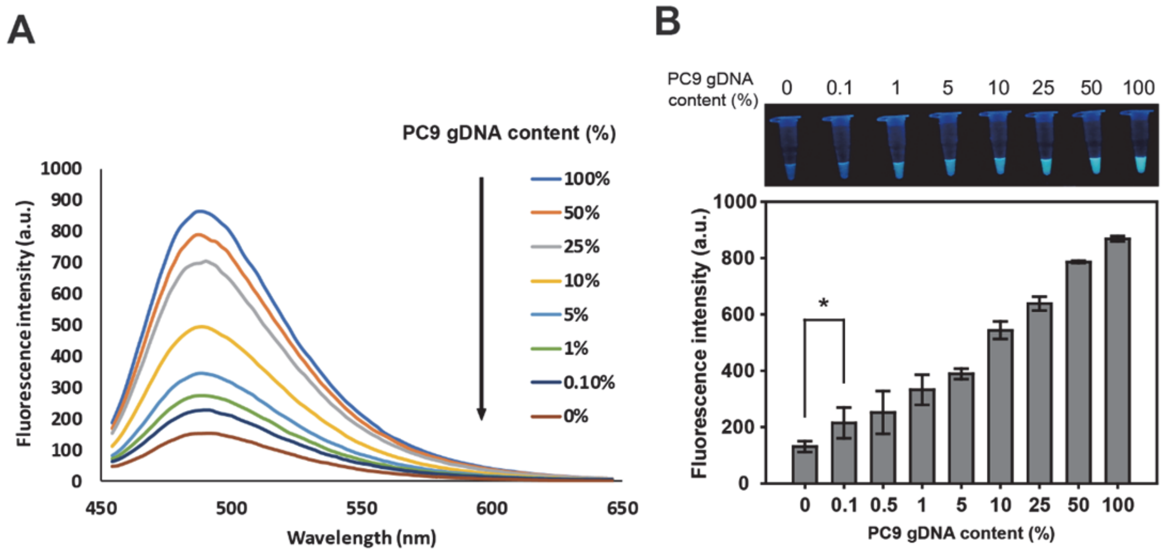

Fig S3. Selectivity of tandem gene amplification assay. (A) Fluorescence emission spectra of ThT (λ_{ex}=430 nm) obtained at different fractions of PC9 gDNA in a fixed amount of gDNA mixtures (50 ng). (B) Bar graphs provide fluorescence intensities at different contents of PC9 gDNA. Statistically significant detection of mutant PC9 gDNA was as low as 0.1% (*, $P < 0.05$ vs. 0%). The data are presented as the mean ± standard deviation of three experiments. Inset image showed the RCA product visualized under UV light.
